# Influence of coolant multi-jets on heat reduction of nose cone with blunt spike at hypersonic flow

**DOI:** 10.1038/s41598-022-20046-5

**Published:** 2022-09-16

**Authors:** Mehdi Ghanbari, Soroush Maddah, Javad Alinejad

**Affiliations:** Sari Branch, Department of Mechanical Engineering, Islamic Azad University, Sari, Iran

**Keywords:** Aerospace engineering, Mechanical engineering

## Abstract

The importance of the cooling system for the design of the forebody of high-speed vehicles is significant due to severe aerodynamic heating at hypersonic flight. In the present study, injection of multi and single-coolant jets on the thermal performance of forebody design of nose cone with the cut spike is thoroughly investigated at hypersonic flow. A three-dimensional model of the blunt cone is presented for computational investigations of proposed jet and spike configurations. Injection of two coolant gasses (Helium and carbon dioxide) into the cooling system of the nose cone with a blunt spike is investigated. Three locations for both opposing and lateral jets are compared to find the efficient jet location. Our results indicate that a single lateral jet injected from the tip of the spike is more efficient for heat reduction. A comparison of the multiple injection system also shows that the heat reduction of the helium gas is about 15% more than CO_2_ jets.

## Introduction

The importance of the forebody design for developing of the current hypersonic vehicle is undeniable and unavoidable^1,2^. The formation of a strong compression shock wave results in the heat source burning the nose of the high-speed vehicles this phenomenon is known as Aerodynamic heating which has a severe disadvantageous on the performance of supersonic vehicles and the management of the design for the body is highly important since these two factors are connected to each other^3,4^.

To resolve high heat production by aerodynamic heating, several techniques and devices have been introduced by different researchers and laboratories initial sharp nose was expected for high-speed vehicles due to burning of the nose sharpness is replaced by blondes while this change results in high drag force on the overall the structure of supersonic vehicles^5,6^. To resolve this issue, the mechanical spike is mounted at the tip of the nose cone and this reduces the drag force although this combination is a big step for the development of the forebody design^7,8^. There is a serious challenge in reducing existing drag and heat protection due to aerodynamic heating^9–11^.

Several computational and experimental reports were published and presented to understand and recognize the physics of hypersonic flow encountered forbody of high-speed vehicles^12–15^. These studies disclosed new aspects of aerodynamic heating nearby the nose of hypersonic vehicles. Most of these researchers have tried to analyze the performance of different devices for the reduction of heat and drag on the main body of hypersonic vehicles^16,17^. Currently, researchers have used numerical techniques to perform an initial evaluation of new conceptions since the computational cost is significantly lower than experimental ones^18,19^.

There are three main concepts for decreasing heat and drag force on the forebody of the nose: opposing jet, mechanical is spike energy decomposition^20,21^. Among these devices, the mechanical spike is now an active and practical method that is widely used in the shuttle and spacecraft^22^. Due to these advantages, this model is developed by the addition of an aerodrome at the tip of a spike, and this change transfers attached to the attachment, and consequently flow temperature in the vicinity of the spike becomes lower in this model^23,24^. To improve the thermal performance of the spike, several modifications had been performed and examined on the spike. injection of opposing and lateral jets is also done to decrease the heat rate nearby the nose of the main body^25,26^. This model is efficient due to the formation of multiple circulations which deflect the incoming flow from the main body and reduce the strength of shark interaction on the shoulder of the main body to develop this mother injection of a coolant cast could help and improve the cooling performance of this technique^27–30^.

**Figure 1:**
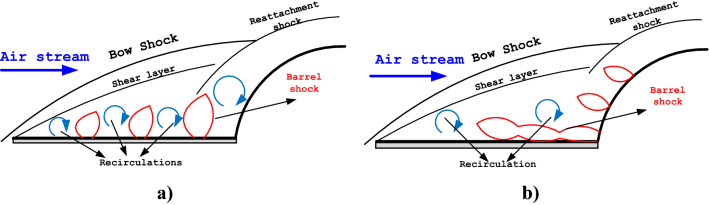
Schematic of the flowfield around the spiked nose cone with (**a**) lateral (**b**) opposing multi-jets.

According to previously mentioned investigations^27,29,31^, it could be found that the spike role is mainly on the reduction of the drag force on the main body. Although heat transfer due to aerodynamic heating is not substantially decreased in this method, this technique is the only practical method for the improvement of the heat and drag force applied to the main body^32–34^. Previous works also described that the opposing jet is more efficient for the heat reduction mechanism near the forebody^35,36^. In fact, this method cools this region abruptly and could not be considered for a long period of time since reservoir coolant gas is the main challenge for this concept^37–39^. Due to these challenges, this study has tried to develop a new device that combines both methods of mechanical spike and fluid device. As demonstrated in Fig. 1, the injection of coolant gas from the main body and spike rod is proposed for the efficient reduction of severe heat production in the vicinity of the nose cone. In this concept, the mechanical spike deflects the main supersonic stream via the production of the bow shock and shear layer while coolant gas is injected the decrease the temperature near the main body. The strength of the circulation close to the main body is increased via this injection system and this results in multiple circulations that are favorable for the cooling mechanism near the main body.

In this study, the impacts of single and multiple cooling jets on the heat transfer rate of aerodynamic heating into the main body are fully investigated. This work applied computational fluid dynamics for the modeling of hypersonic flow around the forebody of hypersonic vehicles. Various positions of coolant injection are investigated to find the optimum location for the heat reduction mechanism. Two different gases are examined to evaluate the main effects of gas type on the cooling mechanism for the proposed configuration. A three-dimensional model of the selected geometry is examined in this work.

## Governing equations and computational methodology

OpenFoam open-source software is applied for the modeling and simulation of the compressible flow^40^. RANS equations are considered the main governing equations and these equations are coupled with the SST turbulence model^41^. The main details of the governing equations are fully described in the previous articles and these refs are recommended to reviewers. It should be mentioned that the species transport equation must be considered because of the presence of helium and/or carbon dioxide in the coolant injection system^42^.

**Figure 2:**
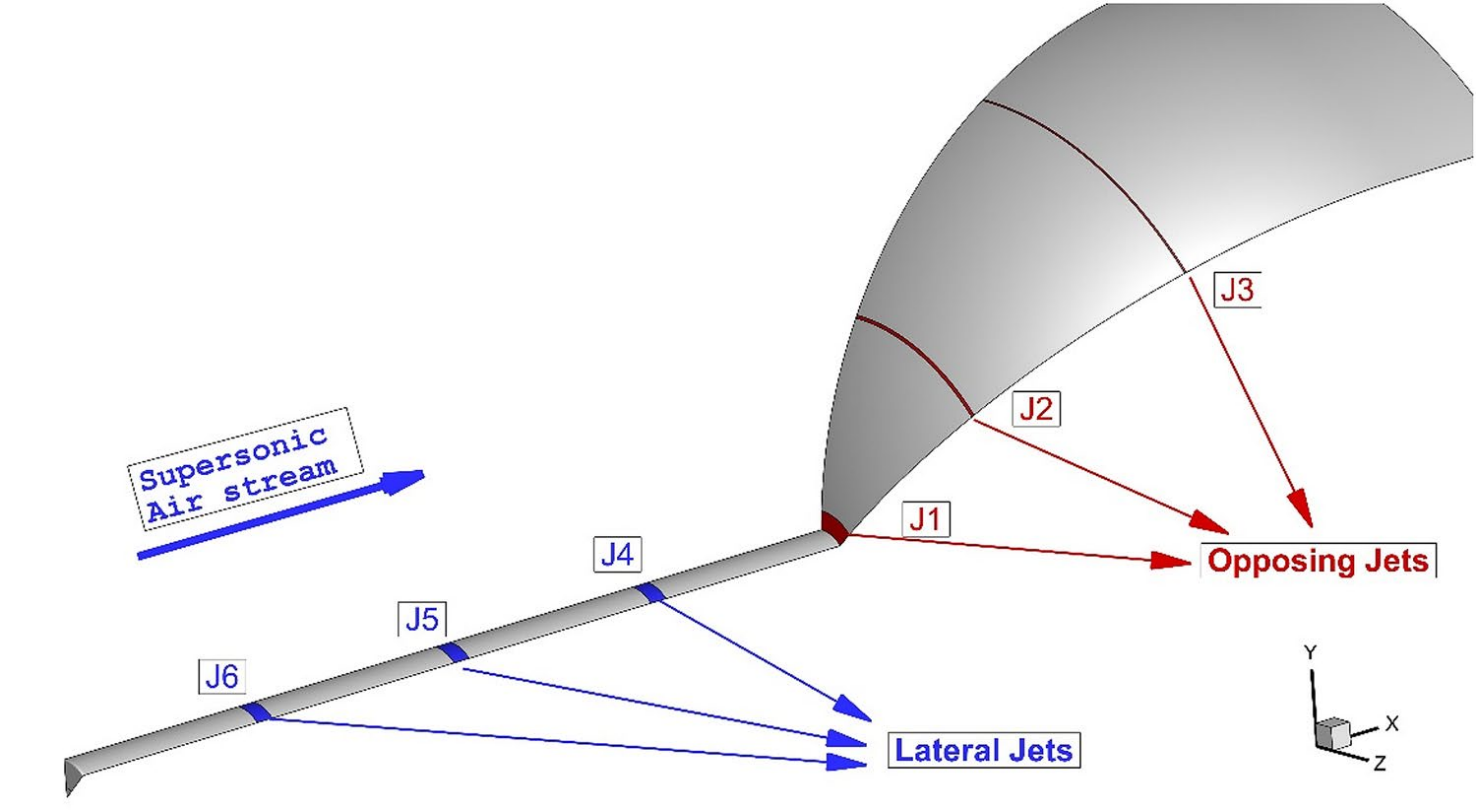
Proposed cooling injection systems.

**Figure 3:**
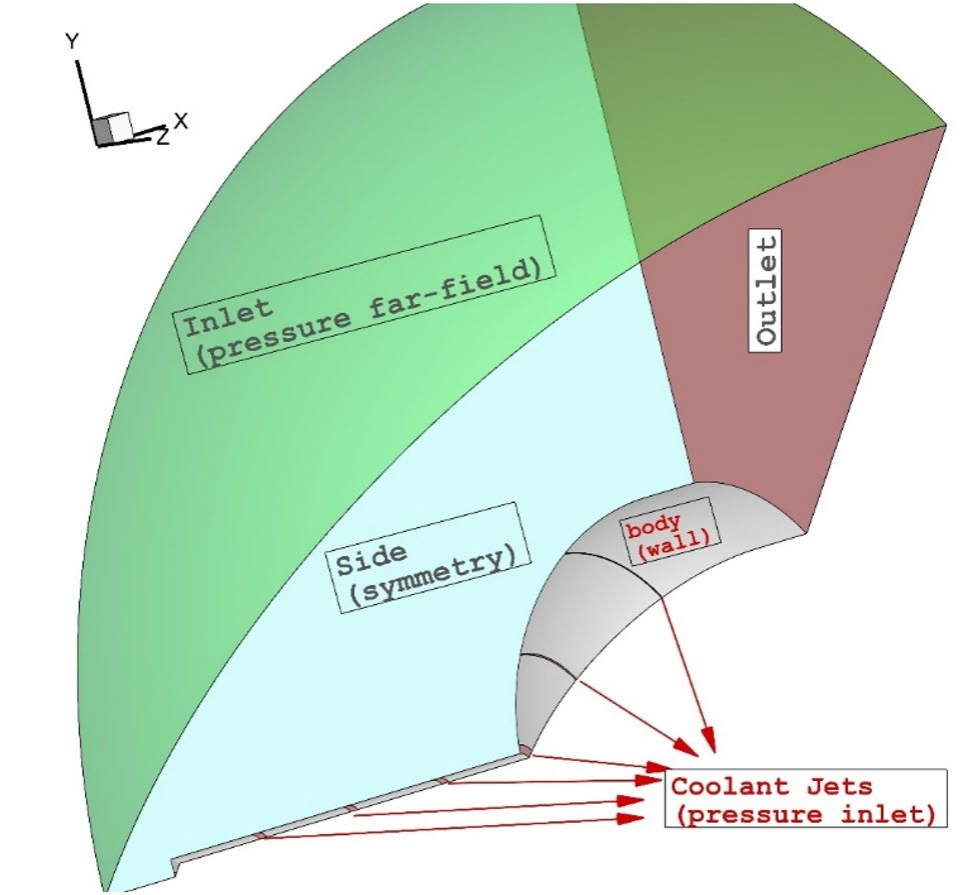
Applied boundary conditions.

Figure 2 demonstrates the geometry and size of the proposed nose cone with a spike. The spike stem and diameter of the forebody are 4 mm and 50 mm, respectively. The lateral and opposing injectors are also determined in this figure. This figure also demonstrates the applied boundary condition. In this work, Mach number, static pressure, and temperature of the free stream are assumed 5, 2550 Pa, and 220 K, respectively^39^. The total pressure of the sonic coolant jet is 10% of the incoming hypersonic flow. Helium and carbon dioxide are the two main coolant gas for this work. The mixing law is used for the calculation of the heat capacity coefficient. The area of all injectors is equal to ensure that the mass flow rate of coolant is constant in different jet locations. As illustrated in Fig. 3, the pressure far-field is applied at the inlet while the outlet is extrapolated from the pressure upstream. To reduce the computational cost, just one-quarter of the model is chosen for the simulations. Besides, faster convergence could be achieved when the inlet condition is chosen as an initial condition^43,44^.

**Figure 4:**
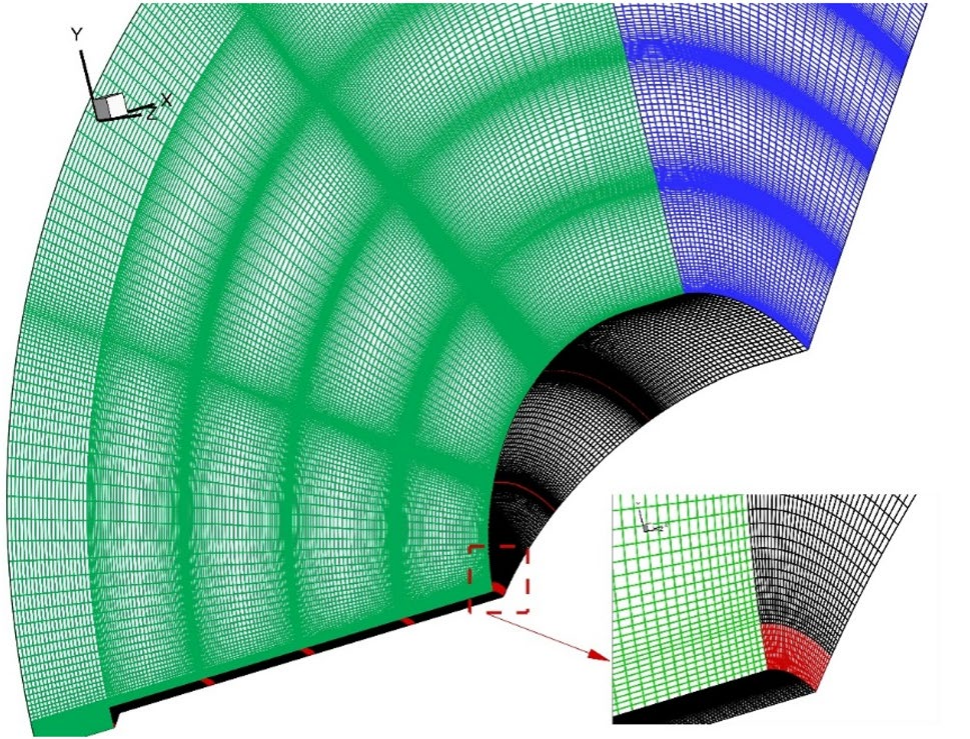
Grid generation.

Several grids are produced for the selected domains to achieve grid-independent results. As depicted shown in Fig. 4, the size of grid elements is smaller in the vicinity of the spike and main body due to the importance of these regions. Besides, main shock interactions occur in these regions and a high-resolution grid is required to improve the convergence of the problem. The details of the produced grids are present in Table 1. The Y + range of the selected grid is less than 6 for our model and this is acceptable for the selected turbulence model. The residual of the converged results is less than 10e− 4. Our solution is converged after 98 h.

**Table 1:** Grid details.

Model	Grid number	Average Stanton numb. on blunt cone (θ = 30)	Average Stanton on numb. blunt cone (θ = 60)
Coarse grid	680,000	0.00212	0.00618
Normal grid	960,000	0.00245	0.00637
Fine grid	1,320,000	0.00251	0.00651
Very fine grid	1,680,000	0.00253	0.00653

## Results and discussion

### Validation

**Figure 5:**
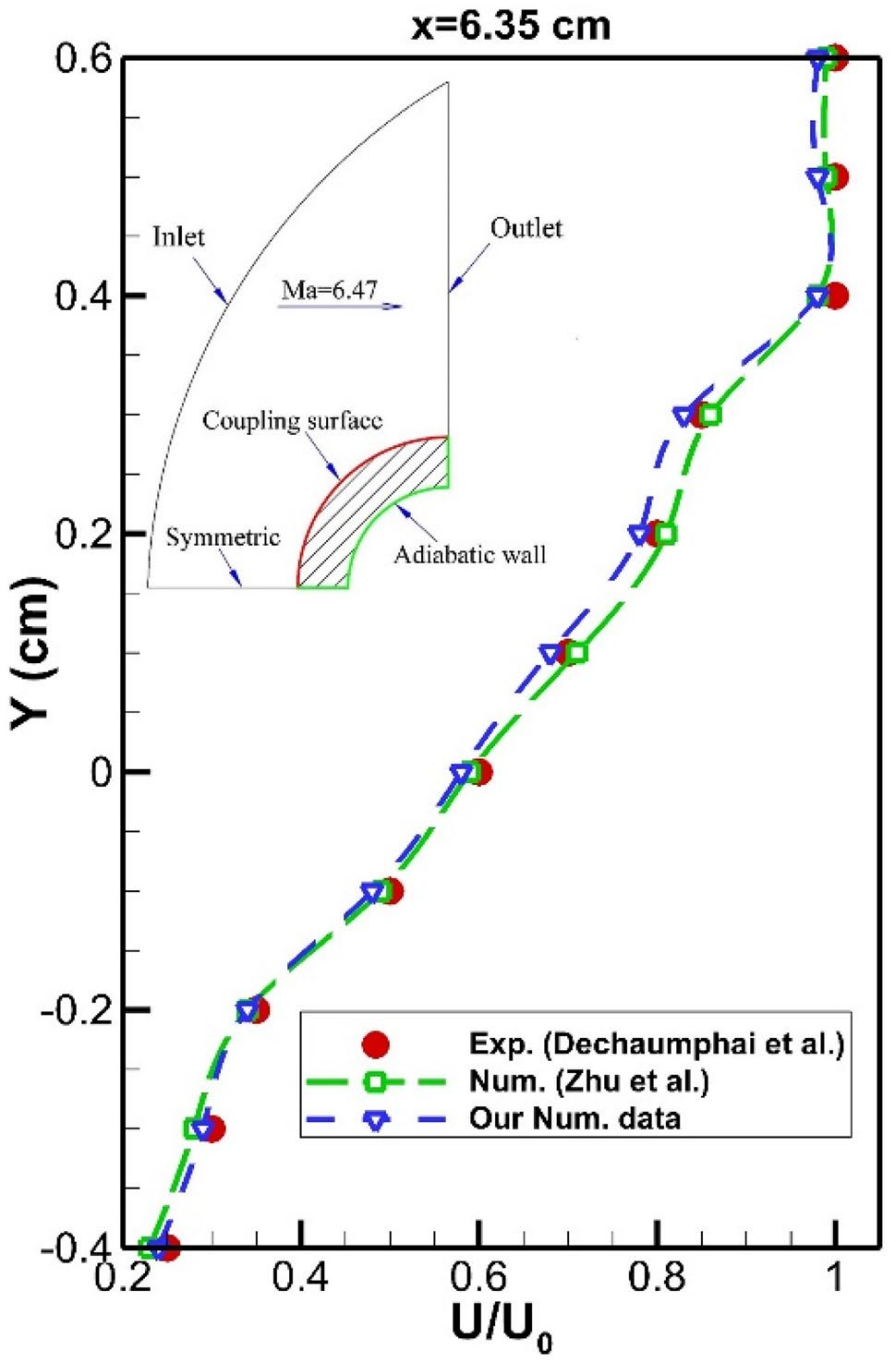
Validation of velocity profile velocity profile on the main nose body.

The validation of our results is done via a comparison of our pressure and velocity profile with those of experimental work. To certify the proposed model and obtained results, we must initially compare our results with experimental data. Since experimental data of the simple cone^44^, we initially simulated the flow characteristic around the nose cone without a spike. Figure 5 demonstrated the velocity profile at a specific location. The numerical data of Zhu et al.^39^ is also presented in these figures for the verification of our results. According to our comparison, the deviation of our numerical simulation with experimental data and other computational studies is within an acceptable range (less than 11%). This deviation is acceptable in modeling of scientific and engineering problems^45–55^

### Double coolant jets

**Figure 6:**
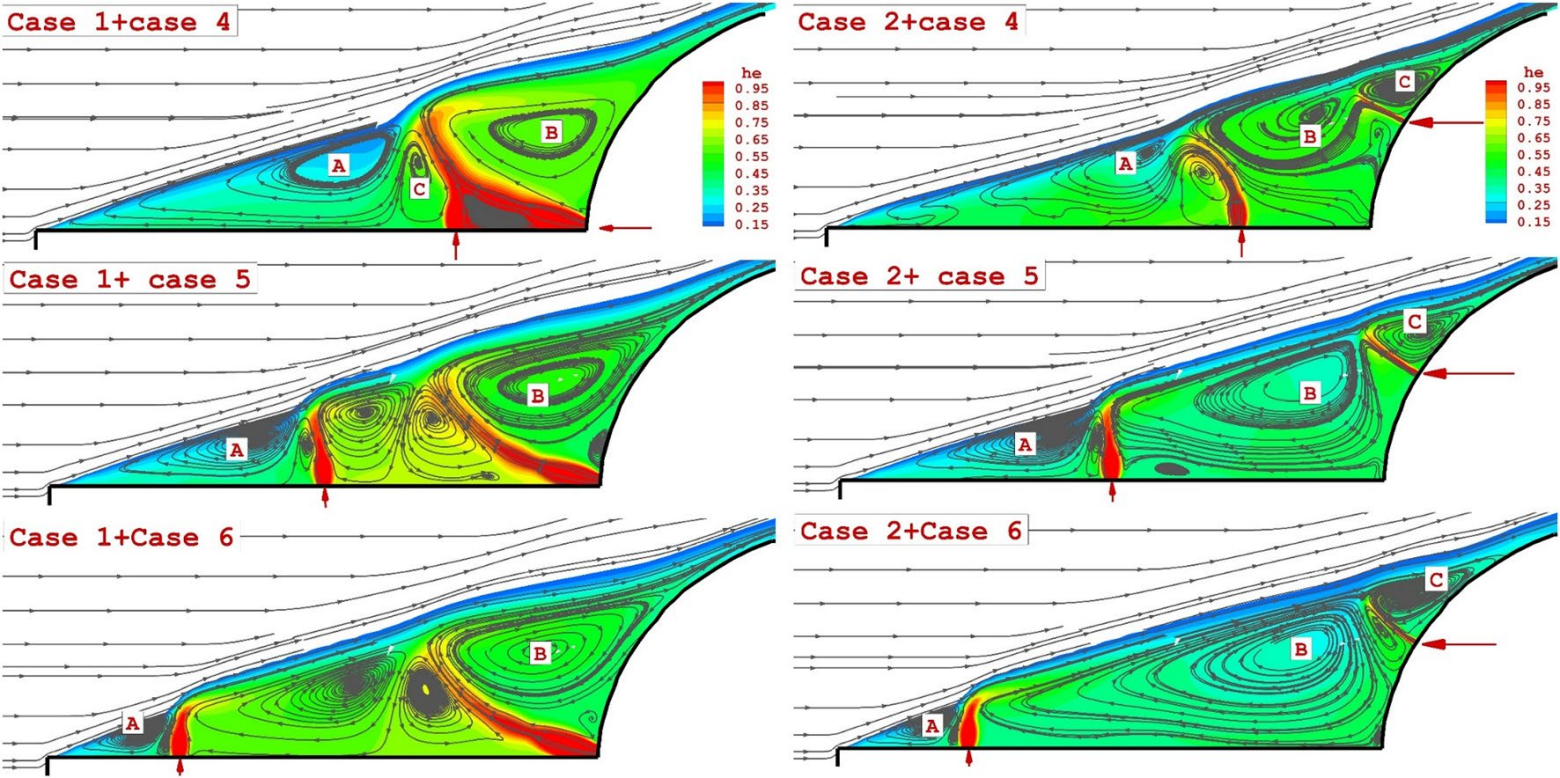
Effects of different arrangement of two helium jets (one lateral jet + one opposing jet) on the flow stream.

The influence of the two helium jets (one lateral jet + one opposing jet) on the flow stream and coolant distribution is demonstrated in Fig. 6. As noticed on the left side of Fig. 6, injection of opposing jet from the tip of nose cone with a lateral jet at different distances indicates that increasing the jet distances increases the circulation and limited the interaction with the main stream.

**Figure 7:**
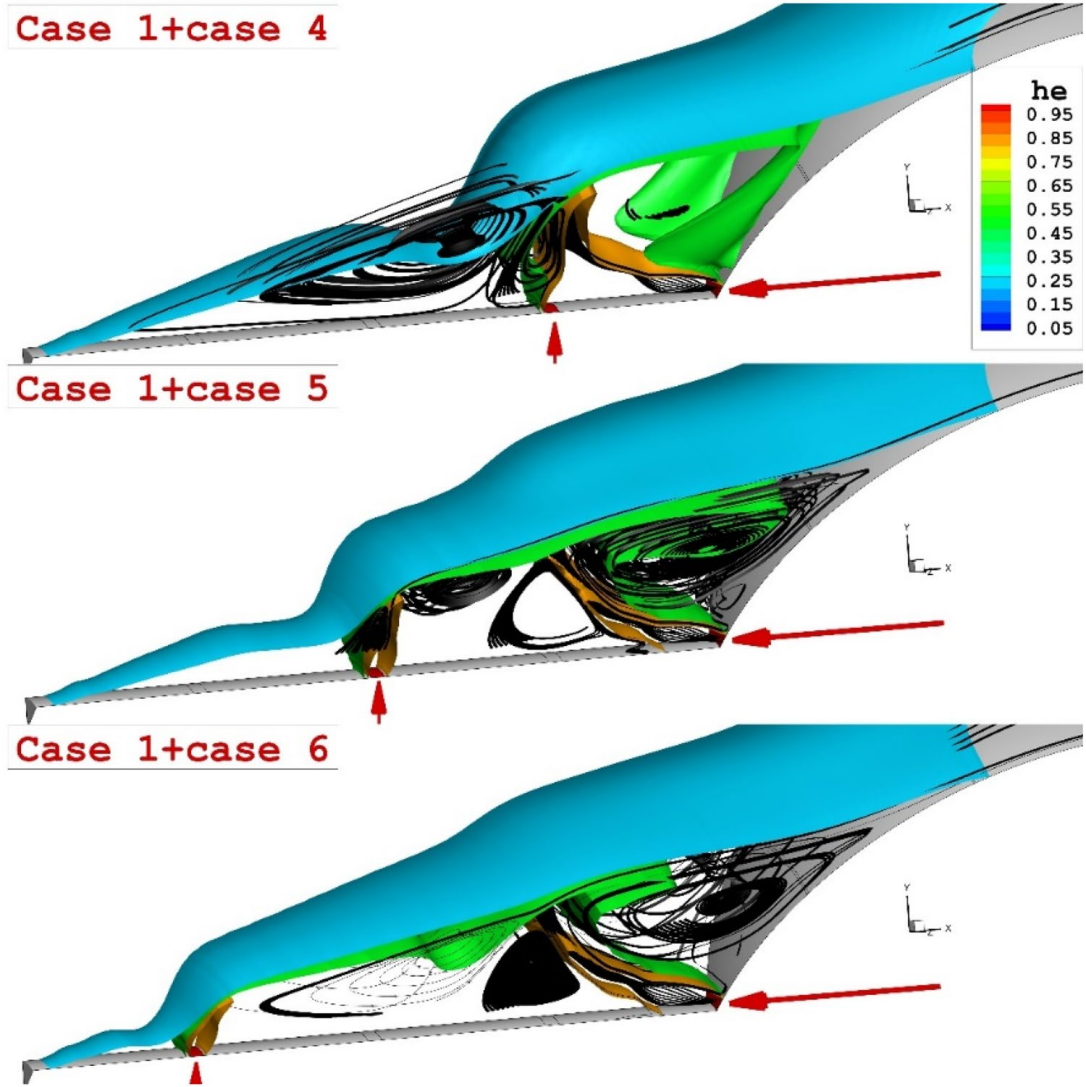
3-D feature of hybrid helium jets around the main body.

As noticed on the right side of Fig. 6, the opposing jet is released at a higher angle of the nose body and displacement of the lateral jet did not change the number of the main circulation in this region. To demonstrate the impacts of two lateral and opposing helium jets, Fig. 7 displays the 3-D structure of the coolant jets and the flow feature of the main stream. Our results certify that the combination of these two injections intensifies the interactions and that would decrease the heat transfer to the main body.

### Triple coolant jets

**Figure 8:**
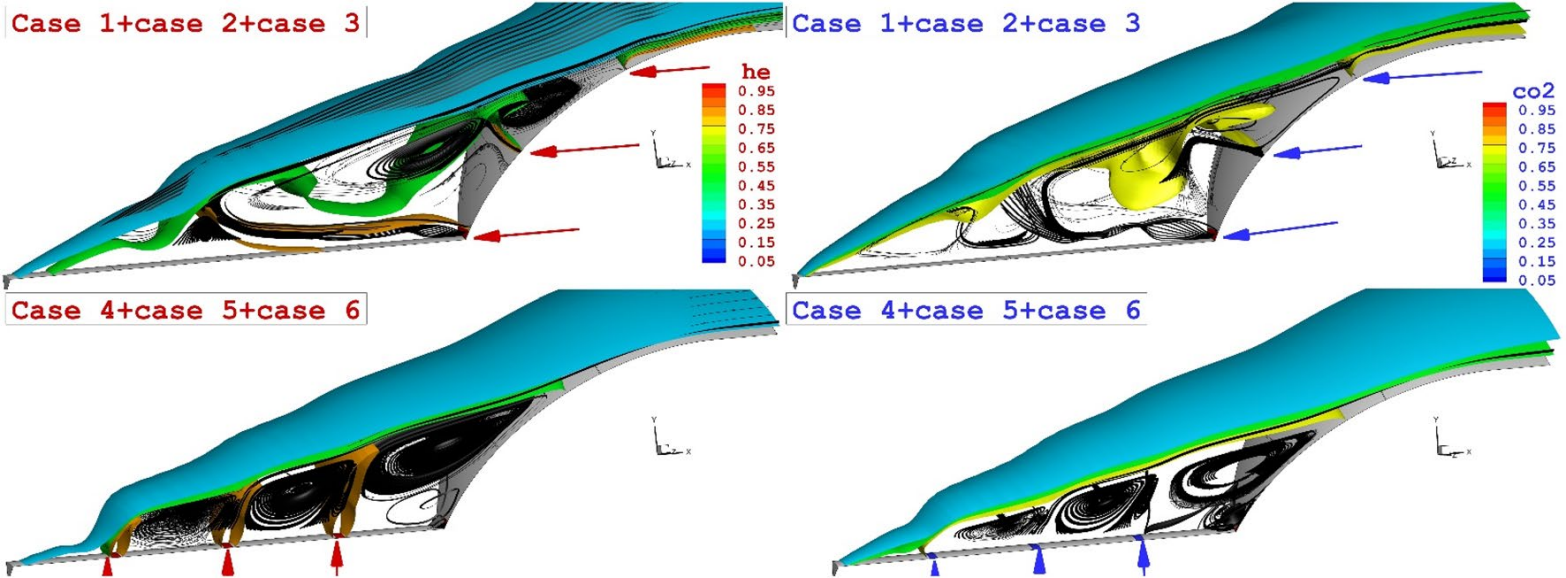
3-D comparison of three helium (left side) and CO_2_ (right side) jet.

**Figure 9:**
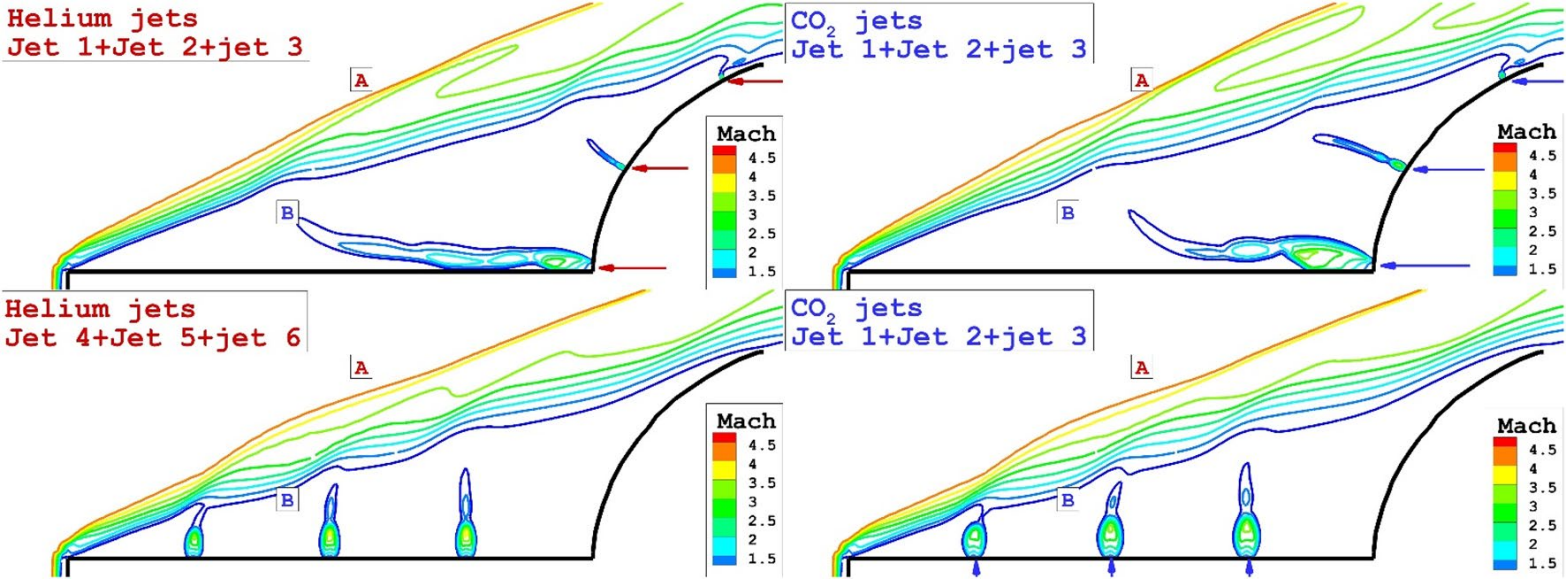
Mach contour of three helium (left side) and CO_2_ (right side) jet in both lateral and opposing jet configurations.

The comparison of the three lateral and opposing jets on the flow feature of hypersonic flow around the spike nose conde is displayed in Fig. 8. Feature of jets and streamline indicate that the triple lateral jets of both helium and CO2 jet are almost similar jet layer while the strength of the stream is not similar. A comparison of the triple opposing jet also confirms that the helium jet could increase the angle of the shear layer as displayed in Fig. 9. The deflection of the shear layer in lateral configuration is visibly discerned in this figure.

**Figure 10:**
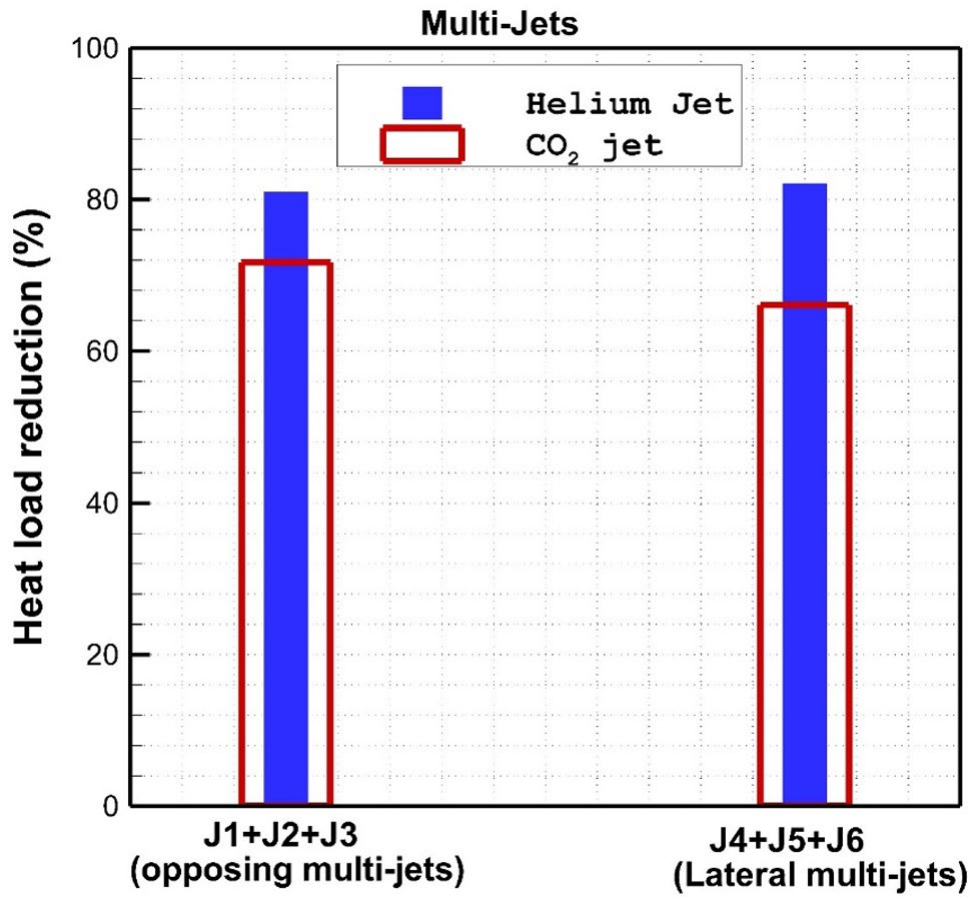
Heat load reduction of helium and CO_2_ jet on the nose cone for different jet locations.

Figure 10 compares the heat reduction performance of these coolant systems for helium and CO_2_ jets. A comparison of the opposing jet indicates that the heat load reduction of the helium jets is about 12% more than the CO_2_ jet. In addition, helium triple jets are more efficient (about 18%) than CO2 ones in lateral injection systems. The main advantage of helium jet is the high diffusivity of this gas in comparison to CO_2_ jet.

## Conclusion

In this work, comprehensive computational studies have been performed to reveal the impacts of lateral/opposing multi-jet on the cooling efficiency of the nose cone with a blunt spike at hypersonic flow. This study applied a 3-D model for the precise estimation of the heat transfer as well as hydrodynamic evaluations. In the first stage, the impact of a single opposing and lateral jet is investigated and the efficient model is determined. Then, the combination of a single opposing jet with a single lateral jet is extensively investigated. Finally, the injection of triple helium and CO_2_ jets is explored to disclose the mechanism of cooling for these jet configurations.

Our results show that the injection of the single lateral jet of helium is more efficient due to the limited interaction of this arrangement. The injection of a double jet also confirms that the performance of this configuration is optimum when the space of these two coolant jets is high. The results of triple jet configurations show that both opposing and lateral jet results in the same heat reduction performance. It is also noticed that the cooling efficiency of Helium triple jets is about 15% more than that of CO2 jets.

## Data Availability

All data generated or analyzed during this study are included in this published article.
